# Comparison of Outcomes between 3 Monthly Brolucizumab and Aflibercept Injections for Polypoidal Choroidal Vasculopathy

**DOI:** 10.3390/biomedicines9091164

**Published:** 2021-09-05

**Authors:** Yoshiko Fukuda, Yoichi Sakurada, Mio Matsubara, Yuka Hasebe, Atsushi Sugiyama, Wataru Kikushima, Kenji Kashiwagi

**Affiliations:** Department of Ophthalmology, Faculty of Medicine, University of Yamanashi, Shimokato 1110, Chuo, Yamanashi 409-3821, Japan; ysugiyama@yamanashi.ac.jp (Y.F.); miom@yamanashi.ac.jp (M.M.); hyuka@yamanashi.ac.jp (Y.H.); asugiyama@yamanashi.ac.jp (A.S.); wkikushima@yamanashi.ac.jp (W.K.); kenjik@yamanashi.ac.jp (K.K.)

**Keywords:** polypoidal choroidal vasculopathy, brolucizumab, aflibercept, intraocular inflammation, resolution of polypoidal lesion(s)

## Abstract

We compared the short-term outcomes between 3-monthly aflibercept and brolucizumab injections for treatment-naïve polypoidal choroidal vasculopathy (PCV). A total of 52 eyes were included. Patients received 3 monthly intravitreal aflibercept (*n* = 38) or intravitreal brolucizumab (*n* = 14). Indocyanine green angiography (ICGA) was performed at baseline and at the 3-month visit. Selection of anti-VEGF agents depended on time. In the brolucizumab-treated group, best-corrected visual acuity (BCVA) improved from 0.27 ± 0.34 (log MAR unit) at baseline to 0.20 ± 0.24 at 3-month visit, which is comparable with the aflibercept-treated group (*p* = 0.87), after adjustment of confounding factors. Central retinal thickness significantly decreased by 43%−44% in both groups. Subfoveal choroidal thickness also significantly decreased by 20.5% during this interval in the brolucizumab-treated group, which was greater than the aflibercept-treated group. The complete resolution rate of polypoidal lesions on ICGA was significantly higher (*p* = 0.043) in the brolucizumab-treated group (78.6%) than in the aflibercept-treated group (42.1%). Intraocular inflammation was observed in 14.3% (2/14) in the brolucizumab-treated group only. In short-term follow-up, intravitreal injection of 3-monthly brolucizumab was comparable with aflibercept in terms of BCVA and morphological improvement along with higher resolution of polypoidal lesion(s) on ICGA.

## 1. Introduction

Polypoidal choroidal vasculopathy (PCV), a variant of type 1 neovascularization secondary to neovascular age-related macular neovascularization (AMD), is characterized by aneurysmal dilation with or without branching vascular networks on indocyanine green angiography (ICGA) [1,2]. PCV accounts for approximately half of advanced AMD according to a clinic-based study undertaken in Japan [3].

Vascular endothelial growth factor (VEGF) is a key factor in the development and progression of neovascular AMD, and intravitreal injection of VEGF inhibitors has revolutionized the treatment of neovascular AMD [4,5]. To date, intravitreal injection of VEGF inhibitors has been the standard treatment for PCV as well as combined therapy involving photodynamic therapy and intravitreal injection of anti-VEGF agents [6,7,8,9]. Currently, in 2021, three anti-VEGF agents are commercially available in Japan: ranibizumab, aflibercept, and brolucizumab. Brolucizumab, an approximately 26 kDa single-chain antibody fragment, is the most recently approved anti-VEGF agent for the treatment of neovascular AMD [10]. In phase 3 HAWK/HARRIER, intravitreal administration of 6.0 mg brolucizumab demonstrated an equivalent visual improvement and a superior morphological improvement compared to intravitreal administration of aflibercept (2.0 mg) [11]. However, to our knowledge, no studies have compared the short-term angiographic outcomes of aflibercept and brolucizumab for PCV. In the present study, we report short-term visual, morphological, and angiographic outcomes for PCV after 3-monthly injections of brolucizumab in comparison with aflibercept.

## 2. Materials and Methods

### 2.1. Participants

Medical charts were retrospectively reviewed for 52 consecutive patients with PCV initiating treatment with 3 monthly intravitreal injections of aflibercept (2.0 mg/0.05 mL, Regeneron Pharmaceuticals, Westchester County, NY, USA) or brolucizumab (6.0 mg/0.05 mL, Novartis Pharma AG, Basel, Switzerland) between January 2018 and February 2021. PCV was diagnosed in the presence of polypoidal lesion(s) on ICGA, regardless of the presence of branching vascular networks. The following inclusion criteria were applied: (1) treatment-naïve eyes with PCV and (2) eyes receiving 3-monthly intravitreal injections of aflibercept or brolucizumab. Eyes with a previous history of uveitis, a previous treatment history for PCV, and with other maculopathy including exudative AMD without evidence of polypoidal lesions on ICGA, were excluded from the study. This retrospective study was approved by the Institutional Review Board of the University of Yamanashi and was conducted in accordance with the tenets of the Declaration of Helsinki. Written informed consent was obtained from each patient prior to the initiation of treatment.

### 2.2. Follow-Up

At the initial visit, all patients underwent a comprehensive ophthalmic examination, including measurement of best-corrected visual acuity (BCVA) on a Landolt chart, intraocular pressure, slit-lamp biomicroscopy with a +78 Diopter lens, color fundus photography, fluorescein angiography, and ICGA using a confocal laser scanning system (HRA-2; Heidelberg Engineering, Dossenheim, Germany), and spectral domain optical coherence tomography (SD-OCT) examination (Spectralis version 5.4 HRA + OCT). OCT scans were performed using 25 horizontal B-scans spanning 20° × 25°. All patients were followed-up monthly. On the 1-month or 2-month visit, a comprehensive examination, excluding angiography, was performed for all patients. At the 3-month visit, the comprehensive examination performed at baseline was repeated. Monthly intravitreal injections of aflibercept (2.0 mg/0.05 mL) or brolucizumab (6.0 mg/0.05 mL) were performed at baseline and 1-month and 2-month visits. The choice of VEGF inhibitors depended on when the patient presented for treatment; aflibercept was administered from January 2018 to August 2020, and brolucizumab was administered from September 2020 to February 2021.

### 2.3. Statistical Analyses

Statistical analyses were performed using Dr. SPSS for Windows software (SPSS, Tokyo, Japan). Continuous and categorical variables between the two groups were tested using the Mann–Whitney U test and the chi-squared test, respectively. BCVA measured on a Landolt chart was converted into a logarithm of the minimal angle resolution (logMAR) for statistical analyses. A two-way ANOVA was employed to compare the values between 2 groups at multiple points. The paired t-test was used to determine the significance of the difference between the values before and after treatment. Statistical significance was set at *p* < 0.05.

## 3. Results

The study included 52 eyes over the study period. All eyes completed 3-monthly injections of aflibercept (*n* = 38) or brolucizumab (*n* = 14). Table 1 shows the baseline characteristics of the two groups; no differences were observed.

**Figure 1:**
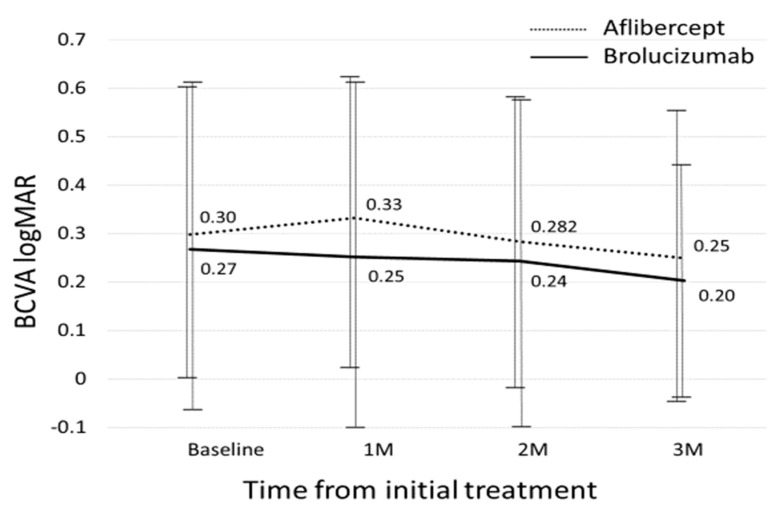
Changes in best-corrected visual acuity between the brolucizumab-treated and aflibercept-treated groups.

**Figure 2:**
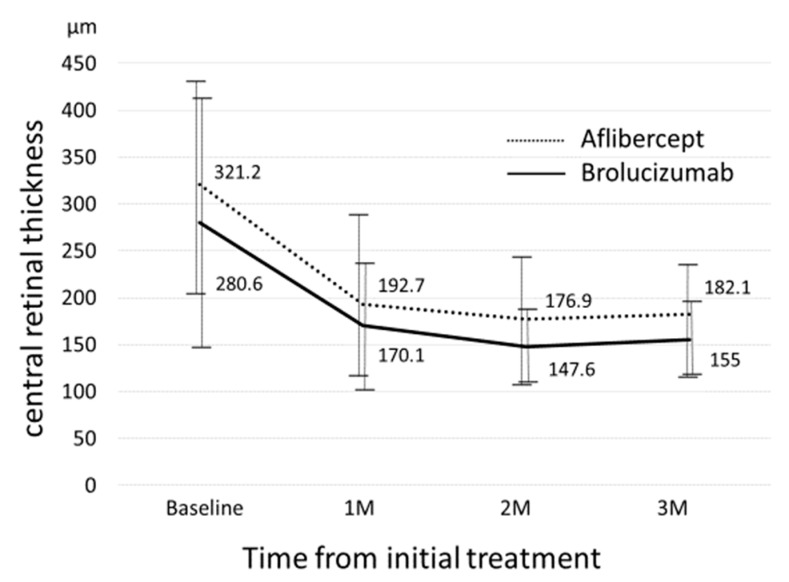
Changes in central retinal thickness between the brolucizumab-treated and aflibercept-treated groups.

**Figure 3:**
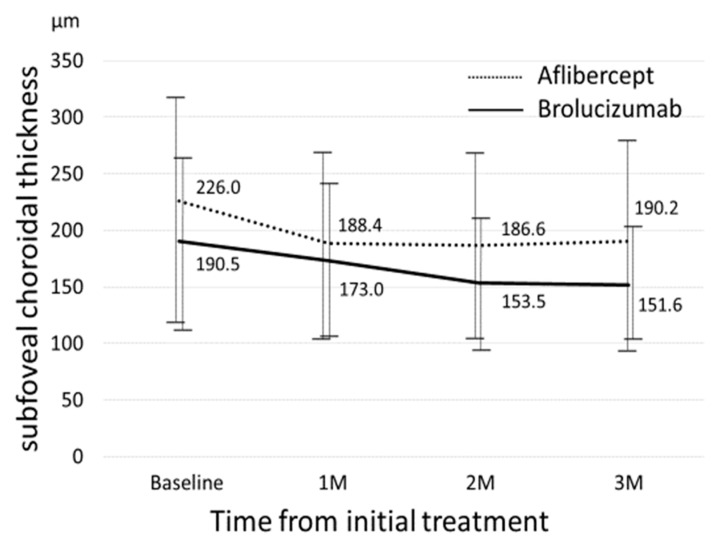
Changes in subfoveal choroidal thickness between the brolucizumab-treated and aflibercept-treated groups.

Figure 1 shows the changes in BCVA between the two groups. Baseline best-corrected visual acuity (BCVA) significantly improved from 0.30 ± 0.30 at baseline to 0.25 ± 0.31 (*p* = 0.013) at the 3-month visit in the aflibercept-treated group and baseline BCVA improved from 0.27 ± 0.34 to 0.20 ± 0.24 at 3 months, which was not statistically significant (*p* = 0.21). Improvement in BCVA was not significantly different at any stage after adjusting for baseline confounders including age, sex, baseline BCVA, central retinal thickness (CRT), and subfoveal choroidal thickness (SCT). Figure 2 shows the changes in CRT between the two groups. In the aflibercept-treated group, baseline CRT significantly decreased from 321 ± 112.8 μm to182.1± 59.5 μm at 3 months (*p* = 4.07 × 10^−10^). In the brolucizumab-treated group, baseline CRT significantly decreased from 280.6 ± 131.7 μm to 155 ± 27.4 μm at 3 months (*p* = 1.0 × 10^−3^). The decrease in CRT was not significantly different between the groups at any stage after adjusting for baseline confounders. Figure 3 shows the changes in SCT between the two groups. In the aflibercept-treated group, baseline SCT significantly decreased from 226.0 ± 100.2 μm to 190.2 ± 96.2 μm at 3 months (*p* = 1.51 × 10^−6^). In the brolucizumab-treated group, baseline SCT significantly decreased from 190.5 ± 73.1 μm to 151.6 ± 49.6 μm at 3 months (*p* = 3.0 × 10^−3^). The extent to which SCT decreased was not significantly different at any visit between the two groups after adjusting for baseline confounders.

Baseline best-corrected visual acuity (BCVA) significantly improved from 0.30 ± 0.30 at baseline to 0.25 ± 0.31(*p* = 0.013) at the 3-month visit, but there was no significant improvement in BCVA from baseline to the 1-month and 2-month visits, respectively (*p* = 0.17 and 0.44, respectively) in the aflibercept-treated group. BCVA improved from 0.27 ± 0.34 at baseline to 0.25 ± 0.36, 0.24 ± 0.33, 0.20 ± 0.24 at 1-month, 2-month and 3-month in the brolucizumab-treated group; however, it was not statistically significant (*p* = 0.71, 0.50, and 0.21, respectively). BCVA improvement was not significantly different between the two groups (*p* = 0.87) after adjusting for age, sex, baseline BCVA, central retinal thickness, and subfoveal choroidal thickness.

Mean central retinal thickness (CRT) significantly decreased from 321 ± 112.8 μm atbaseline to 192.7 ± 83.8 μm at 1-month, 176.9 ± 71.0 μm at 2-months, and 182.1± 59.5 μm at 3-months (*p* = 4.66 × 10^−10^, 3.36 × 10^−11^, and 4.07 × 10^−10^, respectively) in the aflibercept-treated group; mean CRT also significantly decreased from 280.6 ± 131.7 μm at baseline to 170.1 ± 66.6 μm at 1-month, 147.6 ± 33.1 μm at 2-months, and 155 ± 27.4 μm at 3-months, (*p* = 1.2 × 10^−3^, 2.6 × 10^−4^, 1.0 × 10^−3^, respectively) in the brolucizumab-treated group. There were no significant differences in CRT at baseline, 1-month, 2-months, or 3-months between the two groups (all *p* > 0.05).

Mean subfoveal choroidal thickness (SCT) significantly decreased from 226.0 ± 100.2 μm at baseline to 188.4 ± 83.6 μm at 1-month, 186.6 ± 84.8 μm at 2-months, and 190.2 ± 96.2 μm at 3-months (*p* = 1.73 × 10^−5^, 6.81 × 10^−6^, and 1.51 × 10^−6^, respectively) in the aflibercept-treated group, mean SCT also significantly decreased from 190.5 ± 73.1 μm at baseline to 173.0 ± 68.8 μm at 1-month, 153.5 ± 58.0 μm at 2-months, and 151.6 ± 49.6 μm at 3-months (*p* = 1.0 × 10^−2^, 2.63 × 10^−4^, and 3.0 × 10^−3^, respectively) in the brolucizumab-treated group. There were no significant differences in SCT at baseline, 1-month, 2-months, or 3-months between the two groups (all *p* > 0.05).

**Figure 4:**
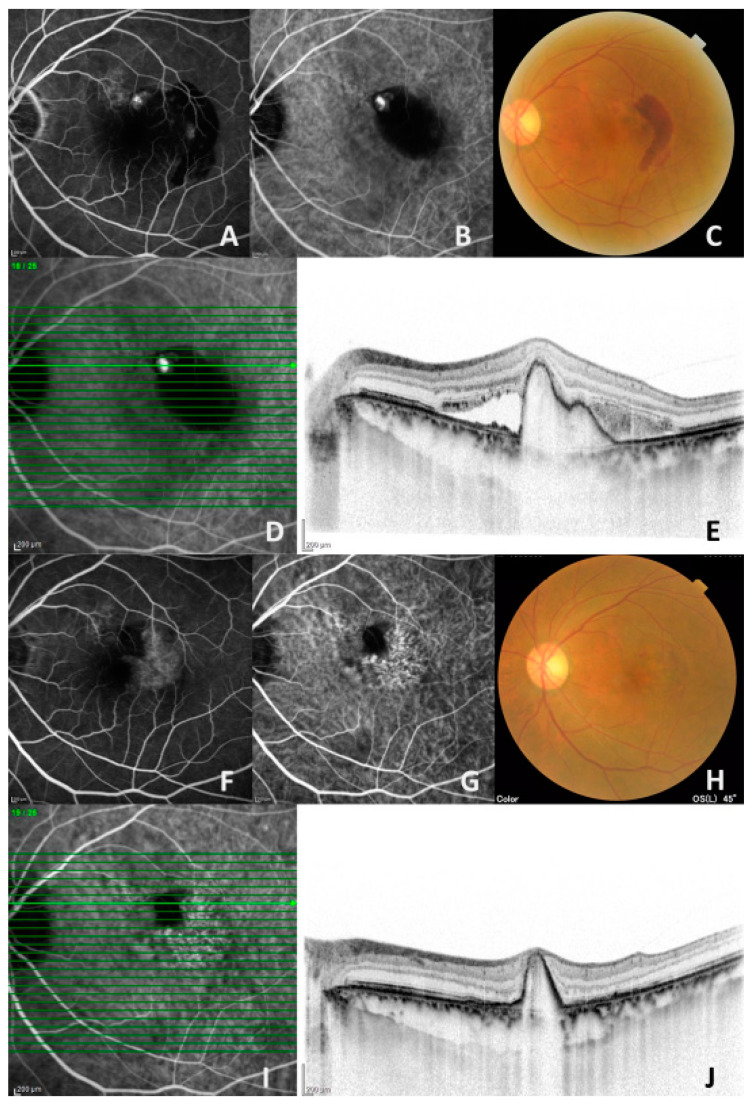
A representative case (76-year-old man) treated with a 3-monthly intravitreal injection of brolucizumab. (**A**,**B**) Fluorescein angiography (left) and indocyanine angiography (ICGA) (middle) demonstrated a hyperfluorescent spot corresponding to a polypoidal lesion at baseline. (**C**) Color fundus photography showed an orange-red lesion and subretinal hemorrhage before treatment. (**D**,**E**) Horizontal optical coherence tomography (OCT) demonstrated retinal pigment protrusion with subretinal fluid and hemorrhage corresponding to a polypoidal lesion on ICGA at baseline. (**F**–**H**) Fluorescein angiography (left) showed staining on the temporal side at the 3-month visit. The polypoidal lesion disappeared on ICGA at the 3-month visit (middle). Subretinal hemorrhage also disappeared on color fundus photography at the 3-month visit (left). (**I**,**J**) On a horizontal OCT scan corresponding to an original polypoidal lesion, retinal pigment epithelial protrusion remained without subretinal fluid.

Table 2 shows the differing prevalence of subretinal fluid (SRF) between the two groups. At all visits, the prevalence of SRF was lower in the brolucizumab-treated group than in the aflibercept-treated group, although this difference was not statistically significant. The complete resolution rate of polypoidal lesions on ICGA was significantly higher (*p* = 0.043) in the brolucizumab-treated group (78.6%, 11/14) than in the aflibercept-treated group (42.1%, 16/38). A representative case with complete resolution of the polypoidal lesion is shown in Figure 4.

**Figure 5:**
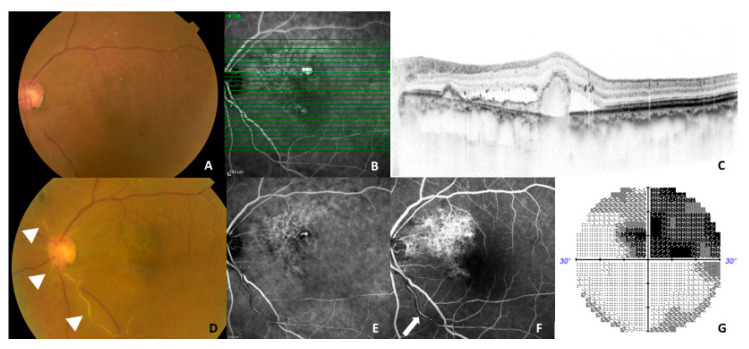
A representative case (77-year-old woman) showing an adverse event during the follow-up. (**A**–**C**) At baseline, fundus photography showed an orange-red lesion superior to the fovea (left), a horizontal optical coherence tomography (OCT) scan demonstrated a double layer sign and retinal pigment epithelial protrusion (left) corresponding to the branching vascular network and a large polypoidal lesion on indocyanine angiography (ICGA) (middle). Whitened retinal vessels as indicated triangles were seen on color fundus photography (**D**) and a residual polypoidal lesion was seen on ICGA. (**E**) Partial perfusion as indicated a white arrow was seen in the inferior arteriole arising from the optic disc on fluorescein angiography 1 month after the third brolucizumab injection. (**F**) Almost half of the superior visual field defects were detected on the Humphrey visual field analyzer 30-2 program. (**G**) BCVA in the left eye was maintained at 0.7, in the decimal format, as was the baseline BCVA.

Intraocular inflammation was noted in two eyes (14.3%, 2/14) in the brolucizumab-treated group. One was in a 63-year-old man, and the other was in a 77-year-old woman. One patient developed anterior segment inflammation with ciliary hyperemia after the second injection. Topical 0.1% betamethasone sodium phosphate was started four times daily, and these findings were immediately resolved. The other developed arteriolar occlusion after the third injection (Figure 5). The patients returned to the clinic four weeks without any symptoms after the onset of this adverse event. Therefore, we could not treat the appropriate prompt treatment. Therefore, visual field loss remained in her right eye.

## 4. Discussion

Several investigators have already reported on brolucizumab treatment, including clinical trials and real-world studies [11,12,13,14]; however, there have been no reports comparing morphological and angiographic outcomes between 3-monthly intravitreal injections of aflibercept and brolucizumab for treatment-naïve PCV. To the best of our knowledge, this is the first report showing the short-term angiographic outcomes of brolucizumab for the treatment of PCV in comparison with aflibercept in treatment-naïve eyes.

Brolucizumab is the smallest commercially available anti-VEGF agent. Owing to its high solubility, brolucizumab can be concentrated to 120 mg/mL; therefore, 0.06 mg brolucizumab can be administered per each 0.05 mL injection. In a single intravitreal injection, the number of brolucizumab molecules and its binding capacity to VEGF is approximately 11 times greater than that of aflibercept [10], which might provide several advantages regarding morphological and angiographic outcomes when treating PCV. In the present study, all eyes (*n* = 52) exhibited SRF on SD-OCT at baseline. Although the difference was not statistically significant, the rate of complete SRF resolution was higher in the brolucizumab-treated group than in the aflibercept-treated group at all points, and all eyes in the brolucizumab-treated group achieved a dry macula at the 3-month visit. This result is consistent with HAWK/HARRIER, which demonstrated that complete resolution of exudation was significantly higher in the brolucizumab (6.0 mg) group (76.0%) than in the aflibercept (2.0 mg) group (65.5%) at 16 weeks [11]. Morphologically, CRT significantly decreased by 43%−44% from the baseline to the 3-month visit in both groups, and SCT also significantly decreased by 16% and 21% in the aflibercept-treated group and brolucizumab-treated group, respectively. Several studies have reported that SCT decreased by 12%–20% after 3-monthly intravitreal injections of aflibercept [15,16,17,18]. A previous study reported that combination therapy involving photodynamic therapy and intravitreal injection of aflibercept/ranibizumab induced approximately 17% and 20% decreases in SCT at 3-month and 12-month visits, respectively [19]. Considering the effect of choroidal thickness change, 3-monthly intravitreal injections of brolucizumab might be comparable to this combination therapy during short-term follow-up.

The rate of polypoidal lesion(s) resolution on ICGA at the 3-month visit was significantly higher in the brolucizumab-treated group (78.6%, 11/14) than in the aflibercept-treated group (42.1%, 16/38). Recently, Matsumoto et al. reported that the disappearance of polypoidal lesion(s) was seen in 15 (78.9%) out of 19 eyes with PCV on ICGA after 3-monthly brolucizumab injections [12]. As mentioned earlier, brolucizumab has a higher binding capacity to VEGF and a stronger effect on choroidal thickness than aflibercept. This may have contributed to the results of the present study.

Regarding BCVA improvement, there was no significant difference between the two groups, which supports the previous study. In the present study, the mean BCVA at baseline was better than the BCVA criteria of clinical trials. Visual improvement was not statistically significant in the brolucizumab-treated group because of the ceiling effect and small sample size.

In the present study, adverse events were observed only in the brolucizumab-treated group (14.3%, 2/14). As shown in Figure 5, a 77-year-old woman developed retinal arteriolar occlusion with a visual field defect in the left eye. Although the number of patients treated with brolucizumab was small, the figure of 14.3% should not be overlooked. Once an adverse event is confirmed, the ongoing brolucizumab treatment should be stopped, and intensive treatment including topical, sub-tenon injection, and systemic steroids should be considered [20,21,22]. In addition, physicians should sufficiently explain the advantages and disadvantages regarding brolucizumab before initiating treatment.

The limitation of the current study is the retrospective nature of the analysis and the small sample size. Therefore, we cannot draw definitive conclusions regarding the short-term efficacy of brolucizumab for PCV. In addition, we did not perform the power calculation although the baseline characteristics between the two groups were not statistically significant. However, we believe that this study is valuable as a pilot study. Further prospective and large-scale studies are needed to confirm or refute the findings of this study.

In summary, the effects of 3-monthly intravitreal injections of brolucizumab resulted in the similar functional outcomes compared with aflibercept, along with the higher resolution of polypoidal lesion(s) on ICGA. Physicians should be careful of the high rate of adverse events when treating with brolucizumab.

## Figures and Tables

**Table 1:** Baseline demographic and clinical characteristics of patients with polypoidal choroidal vasculopathy between two groups.

	Aflibercept	Brolucizumab	*p*-Value
	(*n* = 38)	(*n* = 14)	
Age(years) 95%CI (confidence interval)	71.3 ± 10.0 69.0–73.7	74.7 ± 7.3 69.0–80.5	0.25
Male 95%CI	27 (71.1%) 56–86.2%	10 (71.4%) 44.4–98.5%	1.0
Presence of subretinal fluid	38 (100%)	14 (100%)	1.0
Mean baseline	0.30 ± 0.30 0.20–0.39	0.27 ± 0.34 0.07–0.47	0.57
log MAR BCVA 95%CI			
Mean baseline CRT(μm) 95%CI	321.2 ± 112.8 284–358	280.5 ± 131.7 205–357	0.16
Mean baseline SCT(μm) 95%CI	226.0 ± 100.2 193–259	190.5 ± 73.2 148–233	0.33
Mean number of polyps 95%CI	2.79 ± 3.2 1.72–3.85	2.29 ± 1.5 1.43–3.15	0.16
Mean maximum diameter of polyp(μm)	393.52 ± 234.6	280.2 ± 127.0	0.15

**Table 2:** Prevalence of subretinal fluid at each visit between two groups.

	Aflibercept	Brolucizumab	*p*-Value
	(*n* = 38)	(*n* = 14)	
Baseline SRF (%)	38 (100%)	14 (100%)	1.0
SRF at 1-month visit (%) 95%CI	21 (55.3%) 38.7–71.8%	5 (35.7%) 7.0–64.4%	0.35
SRF at 2-month visit (%) 95%CI	9 (23.7%) 9.5–37.9%	2 (14.3%) 0–35.3%	0.72
SRF at 3-month visit (%) 95%CI	6 (15.8%) 3.6–27.9%	0 (0%) 0%	0.27

SRF: subretinal fluid.

## Data Availability

We will provide the data if necessary.
